# Introductory Address

**Published:** 1859-12

**Authors:** C. B. Chapman


### INTRODUCTORY ADDRESS,

Delivered before the Class of the Ohio College of Dental Surgery.

BY C. B. CHAPMAN.

GENTLEMEN:—Having appropriated a portion of my time which was not devoted to other employments, searching for incidents connected with the early history of American colleges, partly, in the first instance, in order to contribute to my own pleasure, but later with the view of collecting those fragments into such form as to admit of preservation for future use. I take the liberty to present a portion of these memoranda to you as a not unappropriate subject for an introductory lecture in an institution which is mostly devoted to science. The introductory to my last course of lectures in this college was devoted to the incidents connected with the foundation of Harvard and Yale colleges. This will embrace the incidents, briefly presented, connected with the establishment of a considerable number of the institutions of our country. In the history of these institutions we find much that gives a view of the impulses that actuated our fathers in giving cast to the social and intellectual character of our country. The care bestowed in the foundation of these institutions grew out of the respect for religious principles which pervaded these communities. They were impressed with the fact, that in order to a just appreciation of general education by the people, it was necessary that their clergy, and others who were employed in the various professions and positions of trust, must themselves be educated, in science, art and literature, in order that the people might, in some measure, reap the blessings which these might confer. Each of the American colonies, although founded from different motives of speculative or social policy, was impressed with the value of education, as would appear from their early care in the foundation of institutions of learning.

The College of William and Mary, at Williamsburg, Virginia; was, next to Harvard, the oldest college in the United States. It was chartered in 1692, during the reign of William and Mary, and there were donated to it 20,000 acres of land. Harvard had been founded 34 years before, or in 1658.

In the early settlement of the colony of Virginia, an effort was made to establish an institution of learning. In 1619, the treasurer of the Virginia company received five hundred pounds from an unknown hand, to be applied by the company to the purposes of education of Indian youths in the English language and in the Christian religion. Other sums were at different times contributed, and five thousand pounds was in various ways pledged for the endowment of a college. The plan met with the approbation of the king, who recommended to the bishops to have a collection made in each diocese, and by carrying out this plan, fifteen hundred pounds were added to the fund. The college on its establishment was designed for Indian as well as for English youths. The Virginia company appropriated ten thousand acres of land at Henrico, on

James River, a little below the present site of the city of Richmond.

They entered upon the plan of placing one hundred tenants upon the land, who were to receive half of the produce, while the other half was to be applied to the support of the college. It is estimated that the profits would be five pounds to each tenant, or five hundred pounds for the whole number.

A person by the name of Copeland came out from England, who was to act as president of the college as soon as it was organized. George Thorp was appointed agent, and his first care was to arrange with tenants for the occupancy of the lands. He had hardly commenced operations, when he was slain, together with nearly all his tenants, by the Indians, in the great massacre of 1622.

An attempt to pursue the foundation of this college in 1660, was frustrated by the Royal Governor, Sir William Berkley, who, in answer to questions put by lords of plantations, "thanked God that there are no free schools, or printing, in the colonies, and hopes there will not be these hundred years." Express allusion was made in an act of the assembly in 1660, to the necessity of a supply of the ministry and the promotion of piety, and to the lack of able and faithful clergy in the colony. A charter was obtained from the government in England in 1692, through the agency of Rev. James Blair, who was encouraged and aided by the Lieutenant Governor of the colony. The institution took its name from the royal grantors, who appropriated funds, lands, and a revenue duty on tobacco for its support. Buildings were soon erected, and Mr. Blair became its President. The first building erected at Williamsburg was destroyed by fire in 1705. By the bounty of Queen Anne, and the assistance of the House of Burgesses, united by the exertions of Governor Spotswood, this building was soon restored. In the square in front of this building still stands, in a mutilated condition, though with evidence of its former elegance, a statue of Lord Betetourt, ordered by the colonial government in 1771, in gratitude for his just admin'stration of the government. In 1718, a thousand pounds were granted to the college for the support of ingenious scholars.

Robert Boyle, the philosopher, who died in 1691, left his whole estate, after his debts and legacies should be disposed of by his executors, to such pious purposes as in their discretion they should think fit; but recommended that it should be expended for the advancement of the Christian religion.

Of this income, ninety pounds was appropriated to New England, one-half for the support of two missionaries among the Indians, and the other to be given "to the president and fellows of Harvard College, as the salary of two ministers to teach the said natives, in or near the said college, the Christian religion." The remainder of the income was given to the College of William and Mary, on condition of their supporting one Indian scholar for every fourteen pounds received. A house was erected for this purpose on the grounds at Williamsburg, as a school for Indian boys, and the residence of their masters, which still bears upon it the date of 1723. "The experience with the Indians in the south does not appear to have varied much from that of Elliot and his friends in the north. Indians were, however, taught here as late as 1774. Hugh Jones, then chaplain of the Assembly, who was also mathematical professor in the college, in a volume entitled "The Present State of Virginia," says of this attempt:—"The young Indians were formerly boarded and lodged in the town, where abundance of them used to die, either through sickness, change of provisions and way of life; or, as some will have it, often from want of proper necessaries and due care taken of them." Many of these, after being educated, returned to savage customs and heathen rites. A few lived as servants among the English, or loitered away their time in lounging and mischief. He adds, "But 't is a great pity that more care is not taken of them after they are dismissed from school; for they have admirable capacities when their tempers are perfectly understood."

In the early history of our country, the instruction of Indian youth was a romance of educational effort, and had many and free contributors.

The first organization of the college was under a body of visitors, a president and six professors. The corporation was entitled, "The President and Professors of William and Mary's College." The college had a representative in the general assembly. The professorships were as follows: two Divinity; one Greek and Latin; one Mathematics; one Moral Philosophy; and one Boyle's Indian Professorship.

The old colonial administration lent its picturesque dignity to the college.

As a quit rent for the land granted by the crown, two copies of Latin verses were every year presented to the Royal Governor. This was sometimes done with great ceremony, the students and professors marching in procession to the palace, and formally delivering the lines.

At the revolution, the endowment of the college underwent great changes. The war put an end to the colonial revenue taxes for the college support, and funds which had been pledged in England were not received. After the revolution, the loss of the old church and state feeling led to the abolishment of the divinity professorships, and others substituted in their places.

On the breaking out of the revolution, one half of the students, among whom was James Monroe, entered the army. After the surrender of Cornwallis, the French troops occupied the college building, and while they had possession, the president's house was burned,—but the French government promptly paid for its rebuilding. The college building was occupied as a hospital, at the same time, and was much damaged and broken up: but the United States Government has never made any remuneration therefor.

The college charter bears date of 1692, and Rev. James Blair was appointed President in the charter itself. He continued in this office till his death, in 1743, a period of fifty years. In 1689, he was appointed commissary for the colony of Virginia, by the Bishop of London, which office he also held till his death. By virtue of this office he had a seat in the Council of State, and received one hundred pounds per annum, as councillor. President Blair was a man of great perseverance and energy. He had to contend with discouragements and difficulties throughout his whole course. He was often opposed and thwarted in his plans by the royal governors, by the council, and, at times, even by the clergy, who were many of them men of questionable character, and the very refuse of the church in England. At one time, a large majority were arrayed against him. They accused him of exercising his office in a stern and haughty manner, but with nothing more. He must have been a man of great purity of character, for in all his contests, his adversaries never reproached him with any immorality.

His remains were deposited in the churchyard at Jamestown, and an inscription, alluding to his life and services, was engraved on his tombstone. But the stone has been broken, and the inscription has been so damaged that it can not be decyphered;—a fit emblem of the transient character of such inscriptions, in comparison with those which exist in the living character of the good citizen.

He left the whole of his library, which consisted mostly of works on divinity, to the colleges. These, with his own notes and manuscripts, remained in the library until the early part of the present year.

While employed in arranging materials, out of which the facts set forth in this discourse have been gleaned, the following appeared in a daily paper: "William and Mary's College, at Petersburgh, Virginia, was destroyed by fire on the morning of the 8th instant, (March.) The library and laboratory perished with the building. This was one of the oldest colleges in the country."

There is something peculiarly sad in the destruction of a valuable library, especially one which belongs to an old institution of learning. It seems reasonable that every thing which human foresight can suggest, should be practiced in order to secure such against the casualties of fire.

Among the graduates of this institution, four have been Presidents of the United States. These were Jefferson, Madison, Monroe and Tyler; also, Chief Justice Marshall and Gen. Scott.

The College of New Jersey, which is generally known as Princeton College, seems to have succeeded to the decline of the school which was established by Rev. Mr. Tennant, at Neshaming, and known as "The Log College." At this institution, several eminent persons were educated.

A charter was obtained in 1746, and the college was opened at Elizabethtown with Rev. Jonathan Dickinson as first President; but he died in 1747, within a year of its organization.

A new charter was obtained in 1748, and Rev. Aaron Burr, who was father of the politician and statesman, as President. The college was then opened at Newark, during the life of President Burr, but was removed to Princeton in 1757, immediately after his death. President Burr was succeeded by Jonathan Edwards, who was his son-in-law, but he died before he had fully entered upon the duties of his office. The college, which was first erected here, was called Belcher Hall, on account of valuable aid which had been furnished for its erection by Governor Belcher, but he chose to relinquish his claim to that of Nassau Hall, in honor of the great Protestant hero under William III.

Rev. Samuel Davies, succeeded him in 1758. He was a friend of Tennant, with whom he had visited England, and profited by his exemplary life and rich scholarship. President Davies was a finished scholar and ardent missionary; but the college was deprived of his services, by his death, within two years from the time of his appointment. He left some early discourses which made him a valuable name. Among them was one entitled, "Religion and Patriotism the Constituents of a good Soldier," which was suggested by the defeat of Braddock, in which was portrayed with prophetic force, the character and heroism of Col. George Washington; of whom he says, "I cannot but hope that providence has hitherto preserved in so signal a manner for some important service to his country."

Rev. Samuel Finley succeeded President Davies, but died in Philadelphia, in 1766, and his remains were borne to the grave by eight members of the senior class, in accordance with his own request.

Dr. Witherspoon was inaugurated in 1768, and immediately enlarged the field of study in the college by promoting the study of Mathematics and Mental Philosophy. During the Revolution, President Witherspoon was transferred to Congress.

Immediately after the battle at Princeton, in 1777, the college became the scene of conflict between the British occupants and a portion of the army of Washington. In the chapel, in Nassau Hall, hung a portrait of George II, which was destroyed by an American cannon-shot passing through the canvas. In the same frame now hangs a portrait of Washington, which was purchased with fifty guineas, which he gave to the college. This portrait was painted by Peele. The British army plundered and destroyed the library, some of the books having been found in North Carolina, where they were left by the troops of Cornwallis.

There is a picture of the college as it stood at the opening days of the revolution, by John Adams, in his diary, under date of August 26th, 1774, while the young lawyer was on his way to the Continental Congress.

He says, "the college is conveniently constructed; instead of entries across the building, the entries are from end to end, and the chambers are on each side of the entries. There are such entries, one above another, in every story. Each chamber has three windows; two studies with one window to each, and one window between the studies, to enlighten the chamber. Mr. Easton, the Professor of Mathematics and Natural Philosophy, showed us the library. It is not large, but has some good books. He then led us into the apparatus room. Here was a most beautiful machine, an Orrery or Planatarium, constructed by Mr. Rittenhouse, of Philadelphia. By this time the bell rung for prayers, and we went into the chapel. The President soon came in, and we attended. The scholars sing as badly as the Presbyterians in New York.

"After prayers the President attended us to the balcony, where we have a prospect of a horizon of about eighty miles in diameter."

Dr. Samuel Stanhope Smith, who was a son-in-law of Dr. Witherspoon, became President of the College after the death of Dr. Witherspoon, in 1794, which office he held till, from the infirmities of age, he resigned it in 1812. Dr. Alexander has given an account of the impression which President Smith made on his cotemporaries. Describing his appearance in 1801, at Princeton, he says, "certainly, viewing him as in his meridian, I have never seen his equal in elegance of person or manners. Dignity and winning grace were remarkably united in his expressive countenance. His large blue eyes had a penetration which commanded the respect of all beholders. Notwithstanding the want of health, his cheek had a bright rosy tint, and his smile lighted up the whole face. The tones of his elocution had a thrilling peculiarity, and this was more remarkable in his preaching, when it is well known that he imitated the elaborate polish and satirical glow of the French School."

Dr. John Mclane filled the chair of Chemistry and Natural History, of Mathematics and Natural Philosophy, from 1795

to 1812. He was a native of Scotland, was well versed in mathematical sciences, and occupied a high position before he came to this country, having combated with Priestly, the great champion of Phlogiston.

Notes have been found which indicate that he was the first who taught the true nature of flame.

The present President of Princeton College is his son; also, Professor George M. Mclane, the author of a valuable work, which has recently been published, entitled, Elements of Somatology.

The principal early benefactors of this college, were Col. Henry Rutgers, of New York, and Mr. Elias Boudinot, who founded a cabinet of Natural History, and bequeathed eight thousand dollars and four acres of ground. Dr. David Hosack, who was one of its alumni, gave a valuable cabinet of minerals.

In the Philosophical Hall are preserved the electrical machine of Franklin, and the Orrery of Rittenhouse, before referred to.

From information found in the early records of Trinity Church, in New York, it may be inferred that as early as 1707, it was the intention of the colonial government, then represented by Lord Cornbury, to provide a site for a college upon the Island of New York. The subject seems also to have attracted the attention of Bishop Berkley, after a plan he had formed for the establishment of an institution in Bermúda had failed. In 1746, funds were raised by a lottery which was authorized by the colonial government. The income from this source amounted to three thousand four hundred pounds, which was placed in the hands of trustees, a majority of whom were members of the Church of England, and in those of the vestry of Trinity Church. Opposition to the Church of England, seems to have thwarted their plans for a time.

A charter for King's College was however granted, bearing date of 31st October, 1754, and Dr. Samuel Johnson was invited to take charge of the institution.

Dr. Johnson had accompanied President Cutler to England for ordination, and he returned to Stratford in the capacity of a missionary of the society for the propagation of the gospel. He would seem to have been conspicuous in the world of letters, for the University of Oxford had conferred upon him the degree of D. D. Dr. Franklin had so highly appreciated his ability and fitness to take charge of an institution, as to be anxious that he should take charge of the University of Pennsylvania. Bishop Berkley who retained an interest in the college, urged that the first care should be to give instruction in the Greek and Latin Classics, both of which he urged should be well taught, as an incentive to good life and morals.

The college was organized in May, 1755, when Trinity Church conveyed to its Governors the land enclosed by Church, Barclay and Murray streets, and the Hudson River. The only condition they made was that the President should always be a member of the Church of England, and that its liturgy should be used in the services of the college. James Joy went over to England associated with Dr. Smith, provost of the college of Philadelphia, and they returned with liberal donations.

The first commencement was held in 1758. The original building, which is the central portion of the present edifice, was completed in 1760. The President, contemplating retirement on account of advanced age, made application to Bishop Lecher, in England, for an associate who might succeed him in office.

The person chosen for this purpose was Miles Cooper, a young graduate of Oxford. Mr. Cooper was but 27 years of age, and, unlike his predecessor, possessed a convivial turn. But the most notable incident in his career, was his espousal of the tory side in the revolutionary contest. Mr. Cooper became so obnoxious to the people, as one of the tory plotters, that in April, 1775, he and his friends received a significant hint from a published letter, which was signed "three millions," to fly for their lives or anticipate their doom by being their own executioners.

"On the night of May 10th of that year, after Hamilton and his young companions had destroyed the guns on the battery, and one of their comrades had fallen, the mob became greatly incensed, and proceeded to expel Dr. Cooper from the college."

Although Hamilton was active in the cause of liberty, he devised plans which admitted the escape of Mr. Cooper from the college in the night time, when he wandered, half dressed, about the shore of the Hudson, till near morning, when he found shelter in the old Stuyvesant mansion in the Bowery, where he passed the day, and at night was taken on board an English ship of war. He kept the anniversary of these events the next year, by writing a poem descriptive of the circumstances, which may be found in the Gentleman's Magazine for July, 1776.

This is said to be a fair specimen of his poetic powers.

Upon the flight of Dr. Cooper, in 1775, Rev. Benj. Moore was appointed President *pro tem.*, but the exercises of the college were soon after entirely interrupted by the revolution. The building was taken possession of as an hospital, and the library, which contained many valuable works from the University of Oxford and from other sources, was removed and nearly destroyed.

There were no graduates from 1776 to 1784.

On the restoration of peace an iron crown which surmounted the cupola was removed, and, by an act in 1784, the name was changed from King's to Columbia College.

The first student who presented himself, after the revolution, was De Witt Clinton; one of the last who left it before the revolution, was Alexander Hamilton. John Randolph appears among the early students after the restoration.

The next President was William Samuel Johnson, son of the first incumbent. President Johnson was a member of Congress in 1787, while it sat in New York, but when it was removed to Philadelphia, he resigned his place and devoted himself entirely to the interests of the college. He retired in the year 1800, on account of the infirmities of age, and returned to Stratford, where he died at the age of 92. Since the commencement of the present century, there has been no marked incident in the history of this college. Its teachers have uniformly been of the highest order, and the institution has been remarkable in sustaining the classic cast which was imparted by its founders from the staid universities of old England.

The property of this college is more valuable than that of any other institution in the country. It owns valuable real estate, though it has not, hitherto, greatly profited by it. It consists of real estate in the 3d ward, in New York, now estimated at more than half a million; and of property in the 19th ward, once occupied as a Botanic Garden, which was granted by the legislature in 1814. This, which is in Fifth Avenue, consists of two acres, and is valued at about four hundred thousand dollars. The income of the college, in addition to these, consists of rents amounting to nineteen thousand dollars, from other property situated in the 3d ward.

*Brown University* is a monument of the energy and faithful adherence to their church, of the Baptist denomination. It originated when the State of Rhode Island was a British Colony, and was founded through the exertions of Mr. James Manning, a Baptist clergyman, who had been educated at Princeton. At first it bore the name of "the College of Rhode Island," but the privilege was conferred in its charter to change the name to that of the most distinguished benefactor, at a subsequent time. The name was changed to

Brown University, in honor of Hon. Nicholas Brown, who greatly assisted the institution by liberal grants as late as some time in the year 1804.

This institution suffered some of the vicissitudes of war, as may be inferred from the fact that its building was made to serve the purpose of a Military Hospital by the French troops, under Rochambeau, about the year 1777. The exercises of the college were so greatly interrupted, that no catalogue was issued between 1777 and 1784.

Among the distinguished presiding officers and instructors, in this institution, none have deserved more honor than the present incumbent, Rev. Francis Wayland, D. D. President Wayland is a native of the City of New York, where he was born in 1796. He was educated at Union College, and studied Theology at Andover Seminary, and was inaugurated as President of Brown University, in 1827. He adopted the plan of teaching his departments, which were *Moral Science*, *Intellectual Philosophy* and *Political Economy*, by means of lectures instead of the use of text books, which led to the arrangement and publication of his valuable works in these departments.

The history of the foundation of institutions of learning in a country, ought to be regarded among its most notable events. The times are faithfully recorded in the history of different periods when human liberty, and the rights to enjoy the produce of individual labor, was quite different, even in the more enlightened countries of the world, than at this time. The time was when kings, princes, and prelates, did not possess the measure of education which may now be enjoyed by every person in whatever condition in life. If the source of this change is not to be found in the diffusion of knowledge, such improvements will be found to correspond in time, date and locality, to the establishment of educational institutions, and the increase of means for the general diffusion of knowledge.

A high standard of education among the few is always accompanied by an unstable government of a country; but the general diffusion of knowledge, or the education of all, tends to the establishment of such forms of government as will give equal liberty to all classes. The power which knowledge confers, diffuses itself into all the departments and pursuits of life. It prepares the man to act well his part in the various employments in which he may engage.

The philosopher's stone, or universal panacea, will no longer be sought by men of science, nor will they waste their time or energies in useless pursuits.

The rulers of a country will waste the energies and treasure of their subjects to little purpose, unless they mature well their plans of government.

The commander of an army will sacrifice his men with but futile results, unless he matures well his plan for a campaign. The value of the strength and valor expended by his men will correspond with the wisdom of the plans which direct their movements.

This is but a representation of the value of time and strength, whether physical or mental, as we observe their results in the various walks of life. Knowledge confers power—power to the statesman, to the civil and military ruler, as well as to the man of science and the artizan.

The knowledge conferred by the labors of a Humboldt, and Miller, are available to all who are engaged in the various departments of science and industry, and such, forms the most useful capital when well applied.

It is our province to unfold by a limited range of the various departments of science; but we are assured that the diligent student will not remain satisfied without the complete attainment of all that pertains to his own pursuits. You have assembled in this college for the purpose of preparing yourselves for the practice of a useful profession; and will you allow me to urge you so to employ these months which you may remain in this place, that it may not justly be said of any that you are not better prepared to engage on your chosen profession, than those on whom these advantages have not been conferred.

